# Regulation of PP2A_C_ Carboxylmethylation and Cellular Localisation by Inhibitory Class G-Protein Coupled Receptors in Cardiomyocytes

**DOI:** 10.1371/journal.pone.0086234

**Published:** 2014-01-27

**Authors:** Michael R. Longman, Antonella Ranieri, Metin Avkiran, Andrew K. Snabaitis

**Affiliations:** 1 School of Pharmacy and Chemistry, Faculty of Science, Engineering and Computing, Kingston University, Kingston-upon-Thames, Surrey, United Kingdom; 2 King's College London British Heart Foundation Centre, Cardiovascular Division, The Rayne Institute, St Thomas' Hospital, London, United Kingdom; Loyola University Chicago, United States of America

## Abstract

The enzymatic activity of the type 2A protein phosphatase (PP2A) holoenzyme, a major serine/threonine phosphatase in the heart, is conferred by its catalytic subunit (PP2A_C_). PP2A_C_ activity and subcellular localisation can be regulated by reversible carboxylmethylation of its C-terminal leucine309 (leu309) residue. Previous studies have shown that the stimulation of adenosine type 1 receptors (A1.Rs) induces PP2A_C_ carboxylmethylation and altered subcellular distribution in adult rat ventricular myocytes (ARVM). In the current study, we show that the enzymatic components that regulate the carboxylmethylation status of PP2A_C_, leucine carboxylmethyltransferase-1 (LCMT-1) and phosphatase methylesterase-1 (PME-1) are abundantly expressed in, and almost entirely localised in the cytoplasm of ARVM. The stimulation of G_i_-coupled A1.Rs with N^6^-cyclopentyladenosine (CPA), and of other G_i_-coupled receptors such as muscarinic M_2_ receptors (stimulated with carbachol) and angiotensin II AT_2_ receptors (stimulated with CGP42112) in ARVM, induced PP2A_C_ carboxylmethylation at leu309 in a concentration-dependent manner. Exposure of ARVM to 10 µM CPA increased the cellular association between PP2A_C_ and its methyltransferase LCMT-1, but not its esterase PME-1. Stimulation of A1.Rs with 10 µM CPA increased the phosphorylation of protein kinase B at ser473, which was abolished by the PI3K inhibitor LY294002 (20 µM), thereby confirming that PI3K activity is upregulated in response to A1.R stimulation by CPA in ARVM. A1.R-induced PP2A_C_ translocation to the particulate fraction was abrogated by adenoviral expression of the alpha subunit (Gα_t1_) coupled to the transducin G-protein coupled receptor. A similar inhibitory effect on A1.R-induced PP2A_C_ translocation was also seen with LY294002 (20 µM). These data suggest that in ARVM, A1.R-induced PP2A_C_ translocation to the particulate fraction occurs through a G_i_PCR-Gβγ-PI3K mediated intracellular signalling pathway, which may involve elevated PP2A_C_ carboxylmethylation at leu309.

## Introduction

The type 2A protein phosphatase (PP2A) is a serine/threonine protein phosphatase that is ubiquitously expressed in all eukaryotic cells and may account for up to 1% of total cellular protein [1]. A significant proportion (∼30%) of PP2A consists as a scaffold (A) subunit complexed with the catalytic (C) subunit to form PP2A_A/C_ heterodimers [2]. The PP2A_A/C_ heterodimer subunits provide a platform for the binding of a third component, the regulatory B subunit, which facilitates “targeting” of the heterotrimeric holoenzyme towards target substrates [3].

The PP2A catalytic subunit undergoes reversible post-translational phosphorylation and carboxylmethylation, both of which can alter catalytic activity and cellular distribution of PP2A. Phosphorylation of threonine304 and tyrosine307 residues in the carboxyl terminus of the protein is associated with inhibition of PP2A_C_ activity [4], [5]. Carboxylmethylation of PP2A_C_ occurs at the C-terminal leucine309 (leu309) and is catalysed by leucine carboxylmethyltransferase-1 (LCMT-1), a member of a large methyltransferase family of enzymes that utilise S-adenosyl methionine (SAM/AdoMet) as a universal methyl donor [6]. The carboxylmethylation of leu309 increases the binding affinity of the PP2A_A/C_ heterodimer for some, but not all, regulatory B subunits, which have been classified into four separate sub families and are encoded by 15 human genes: *PPP2R2/B* (A–D), *PPP2R5/B′* (A–E), *PPP2R3/B″* (A–C) and the *striatins/B′″* (1, 3 and 4). Through alternative gene splicing, several of these genes can generate a number of splice variants, resulting in the expression of 20+ regulatory B subunits [7]. The importance of PP2A_C_ leu309 carboxylmethylation by LCMT-1 for recruitment of regulatory B subunits to the PP2A_A/C_ heterodimer can be considered a sliding scale, whereby it is an absolute prerequisite for *PPP2R2/B* subunit recruitment by the PP2A_A/C_ heterodimer and progressively less important for the recruitment of *PPP2R5/B′*, *PPP2R3/B″* and the *striatin/B′″* subunits to PP2A_A/C_ [8].

The carboxylmethylation of PP2A_C_ on leu309 is reversed by the protein phosphatase methyl esterase PME-1 [9], an enzyme found to be associated with an inactive and demethylated pool of PP2A_C_ subunits [10]. Evidence suggests that PME-1 can displace the two metal ions from the active site that are required for PP2A_C_ activity, thereby inhibiting PP2A_C_ activity in a demethylation-independent manner (6). This inactive pool of PP2A_C_ can also be reactivated by a phosphotyrosyl phosphatase activator (PTPA) *in vitro* [10]. The mechanism of PP2A_C_ reactivation by PTPA remains undefined, however, evidence suggests that the PTPA-mediated peptidyl-prolyl *cis/trans* isomerase activity and consequent conformational change to the structure of PP2A_C_ seems likely [11].

Structural X-ray crystallographic studies [12]–[14] have shown that the last 6 C-terminal amino acids (304TPDYFL309) of PP2A_C_ interact with both the active site and a unique lid domain of LCMT-1, the latter of which may confer some degree of substrate specificity. These 6 C-terminal amino acids of PP2A_C_ are highly conserved between species [15] and occupy the deep active site pocket of LCMT-1, which is facilitated by interaction between the catalytic sites of active PP2A_C_ and LCMT-1, thereby suggesting that PP2A_C_ can only be carboxylmethylated by LCMT-1 once in an active conformation [16]. This ensures that only active PP2A_C_ subunits can be carboxylmethylated and is therefore thought to prevent uncontrolled PPP2R2/B-PP2A-mediated dephosphorylation [16].

The importance of understanding the regulation of cellular PP2A_C_ activity is confirmed by the number of human diseases such as diabetes [17], cancer [18], Alzheimer's disease [19] and heart failure [20], [21] which appear to share (in part) a PP2A-based aetiology. Despite this, the role and regulation of PP2A_C_ carboxylmethylation in cells is poorly understood. In the heart, several studies [22]–[25] demonstrate that stimulation of adenosine type 1 receptors can induce not only PP2A_C_ carboxylmethylation but also PP2A_C_ translocation to the membrane-rich particulate fraction of ventricular myocytes. Furthermore, previous work by ourselves [23] and others [22] has shown that the phosphorylation status of selected proteins that are expressed within the membrane-rich particulate fraction of ventricular myocytes is reduced, thereby altering the function of these proteins. Since type 2A protein phosphatases represent the only identifiable LCMT-1 substrates [26], it is surprising that this important LCMT-1/PME-1/PP2A_C_ intracellular signalling axis has not been more extensively studied in cardiac cells.

Hence, the current study investigates the intracellular signalling mechanisms through which G_i_PCR stimulation regulates PP2A_C_ carboxylmethylation and subcellular distribution in ARVM. We demonstrate that (i) the LCMT-1/PME-1/PP2A_C_/B55α intracellular signalling axis is mainly localised in the cytoplasm, (ii) PP2A_C_ carboxylmethylation can be induced by multiple G_i_PCR agonists, (iii) A1.R stimulation increases the cellular association between PP2A_C_ and LCMT-1 and (iv) A1.R-induced PP2A_C_ translocation to a membrane-rich particulate fraction occurs through a Gβγ - PI3K pathway.

## Materials and Methods

Animal tissue used in this study was obtained in accordance with the UK Home Office Guidance on the Operation of the Animals (Scientific Procedures) Act 1986, published by Her Majesty's Stationery Office, London, and approved by the Institutional Animal Care and Use Committee (IACUC) of King's College London. Healthy animals were sacrificed by a schedule one procedure completed by a home office licensed individual such that animal suffering was categorised as minimal.

### Antibodies and reagents

Antibodies were from the following sources: monoclonal anti-PP2A_Cα/β_ (05-421), monoclonal anti-methyl PP2A_c_ (2A10) and monoclonal anti-demethylated PP2A_C_ (4B7) antibodies were purchased from Millipore/Upstate Biotechnologies, UK; polyclonal anti-Gα_t1_ (K20), monoclonal anti-LCMT-1 (4A4), polyclonal anti-PP2A_C_ (FL-309) and monoclonal anti-PME-1 (B12) antibodies were purchased from Santa Cruz Biotechnology; polyclonal anti-cardiac troponin I (cTnI), monoclonal anti-PKB (2H10), rabbit monoclonal anti-PKB (11E7) and phosphorylated PKB (ser473) were obtained from Cell Signaling Technology. Polyclonal anti-GFP antibody was purchased from Clontech; anti-B55α (PPP2R2A) polyclonal antibody was purchased from Merck Calbiochem; HRP-conjugated donkey anti-rabbit and sheep anti-mouse secondary antibodies were purchased from GE Healthcare, UK. G_i_PCR agonists N^6^-cyclopentyladenosine (CPA), carbachol (CCH) and CGP42112 (CGP) were purchased from Sigma-Aldrich; LY294002 was purchased from Merck Calbiochem. Recombinant adenovirus coexpressing enhanced green fluorescent protein (EGFP) and the Gα subunit of transducin (Gα_t1_) was a kind gift from Professor Thomas Wieland [27], University of Heidelberg, Germany.

### Cell culture of adult rat ventricular myocytes

ARVM were isolated from the hearts of adult male Wistar rats (200–250 g, B & K Universal Ltd) by collagenase-based enzymatic digestion, as previously described [28]. The isolated myocytes were pelleted by brief centrifugation at 50 *g* and washed at room temperature with M199 culture medium (Invitrogen) containing 100 IU/ml penicillin/streptomycin and 10% FBS. Following further centrifugation at 50 *g*, ARVM were resuspended in modified M199 containing (in mM) creatine 2, carnitine 2, taurine 5, 100 IU/ml penicillin/streptomycin and 10% FBS. ARVM were then added to 6-well cell culture plates, that had been earlier coated with laminin as previously described [28] and allowed to adhere for 90 minutes in an incubator (37°C, 5% CO_2_). The culture medium was then replaced with fresh modified M199 medium, prior to adenoviral infection.

### Adenoviral infection of adult rat ventricular myocytes

The recombinant adenovirus which co-expresses enhanced green fluorescent protein (EGFP) with the bovine Galpha subunit of the transducin GPCR (AdV:Gα_t1_) was constructed using the AdEasy system [29] and a kind gift from Professor T Wieland, University of Heidelberg [27], [30], [31]. Adenovirus expressing EGFP alone (AdV:EGFP) was used as a control. The recombinant adenoviruses were amplified in HEK-293 cells and purified over CsCl_2_ gradients as previously described [28], which produced high-titre viral stocks of 3–5×10^9^ plaque forming units (pfu)/ml, as determined by a serial end-point dilution assay [32]. ARVM were infected with AdV:EGFP (control) or AdV:Gα_t1_ at a multiplicity of infection (MOI) of 50 pfu/ARVM (which provided >90% transduction efficiency). The adenovirus-containing medium was then removed after 60 min and replaced with fresh modified M199 medium. ARVM were maintained in an incubator (37°C, 5%CO_2_) and were used for experiments 18 h after infection.

### Stimulation of adult rat ventricular myocytes with G_i_PCR agonists

ARVM were exposed to the angiotensin II type 2 receptor agonist CGP42112 (0.01–100 nM, 10 min), the muscarinic receptor agonist carbachol (0.01–100 µM, 10 min) or adenosine type 1 receptor (A1.R) agonist CPA (0.01–100 µM, 10 min), to stimulate their cognate GPCRs. Control cells received vehicle (phosphate buffered saline or 0.1% DMSO in PBS) for an identical period. At the end of the exposure period, ARVM were lysed with either Laemmli SDS-PAGE sample buffer (for immunoblotting) or a lysis buffer for subcellular fractionation.

### Immunoprecipitation of PP2A_Cα/β_ from adult rat ventricular myocytes

ARVM were exposed to vehicle control (DMSO, 0.1%) or CPA (10 µM) for 10 min and then lysed with ice-cold immunoprecipitation buffer (in mM); imidazole-HCl 20, EDTA 2, EGTA 2 and 0.1% Triton X-100. The culture plates were frozen on a volume of liquid N_2_ and subsequently thawed at room temperature at which point cellular lysates were harvested. The insoluble cellular components were collected as a pellet following centrifugation at 14000 *g* for 10 min at 4°C. A 100 µl aliquot of the supernatant was then removed and to this 50 µl of 3× Laemmli sample buffer was added, this is referred to as the “input”. To immunopreciptate PP2A_Cα/β_, 100 µg of protein from the remaining supernatant was incubated with 5 µg of immunoprecipitating antibody (mouse monoclonal anti-PP2A_Cα/β_) or non-immune mouse IgG (Millipore, UK), overnight at 4°C. Then 50 µl of protein A sepharose beads (GE Healthcare) was added for a further 1.5 hours at 4°C. The beads were collected by centrifugation at 370 *g* for 1 min and at 4°C. The immunocomplex was then washed twice with ice-cold PBS and 50 µl of 2× Laemmli sample buffer was added to the immunocomplex. All samples were then heated to 95°C for 5 min prior to SDS-PAGE and Western immunoblotting. Equal volumes (20 µl) of each sample were loaded per well.

### Subcellular fractionation of adult rat ventricular myocytes

ARVM were fractionated using a previously described protocol [33], with minor modifications. In brief, ARVM were lysed in ice-cold lysis buffer at pH 7.5 containing (in mM) Tris-HCl 50, EGTA 5, EDTA 2, DTT 5, as well as 0.05% digitonin and Mini-Complete protease inhibitor cocktail (Roche, Germany). The samples were then frozen by floating the culture plate on a volume of liquid N_2_ and subsequently thawed at room temperature, at which point cellular lysates were harvested. Cell lysates were then centrifuged at 14000 *g* for 30 min at 4°C and the supernatant, which contained the cytoplasmic fraction, was removed. The pellet, which contained the membrane-rich particulate fraction, was then solubilised in an equal volume of the digitonin-based lysis buffer containing 1% Triton X-100. The determination of total PP2A_c_ content in the cytosolic and particulate subcellular fractions was achieved by NaOH-mediated saponification of the methylated C-terminal leu309 residue of PP2A_C_. In brief, an equal volume of 200 mM NaOH (100 mM final [NaOH]) was added to each cytosolic and particulate fraction, followed by incubation for 30 min at 30°C and subsequent pH neutralisation by the addition of 1 M HCl, as previously described [23]. This ensured that all PP2A_C_ protein in the sample was in a demethylated state, thereby allowing detection of total PP2A_C_ content by the anti-PP2A_C_ monoclonal antibody (4B7). Equal volumes of Laemmli SDS-PAGE sample buffer were added to both fractions and the fractionated proteins resolved by 12% SDS-PAGE followed by western immunoblotting.

### Western immunoblotting

Western immunoblotting was carried out as previously described [34]. In brief, protein samples were separated by 12% SDS-PAGE, transferred to PVDF or nitrocellulose membranes where appropriate and probed with the primary antibodies as described earlier. Primary antibodies were detected by appropriate donkey anti-rabbit or sheep anti-mouse secondary antibodies linked to horseradish peroxidase (GE Healthcare, UK). Specific protein bands were detected by enhanced chemiluminescence (GE Healthcare, UK) and band intensity was quantified using a calibrated densitometer (GS-800, Bio-Rad) and Quantity One® 1-D analysis software (v 4.6.2).

### Statistical analysis

Data are presented as mean ± SEM. Data were subjected to ANOVA (GraphPad Prism v6.0.1) and if a significant difference (p<0.05) was detected, further analysis by a Dunnett's modified t-test (for comparison of each group with a single control) was performed.

## Results

### Compartmentalisation of the PP2A_C_, LCMT-1 and PME-1 signalling axis in adult rat ventricular myocytes

**Figure 1:**
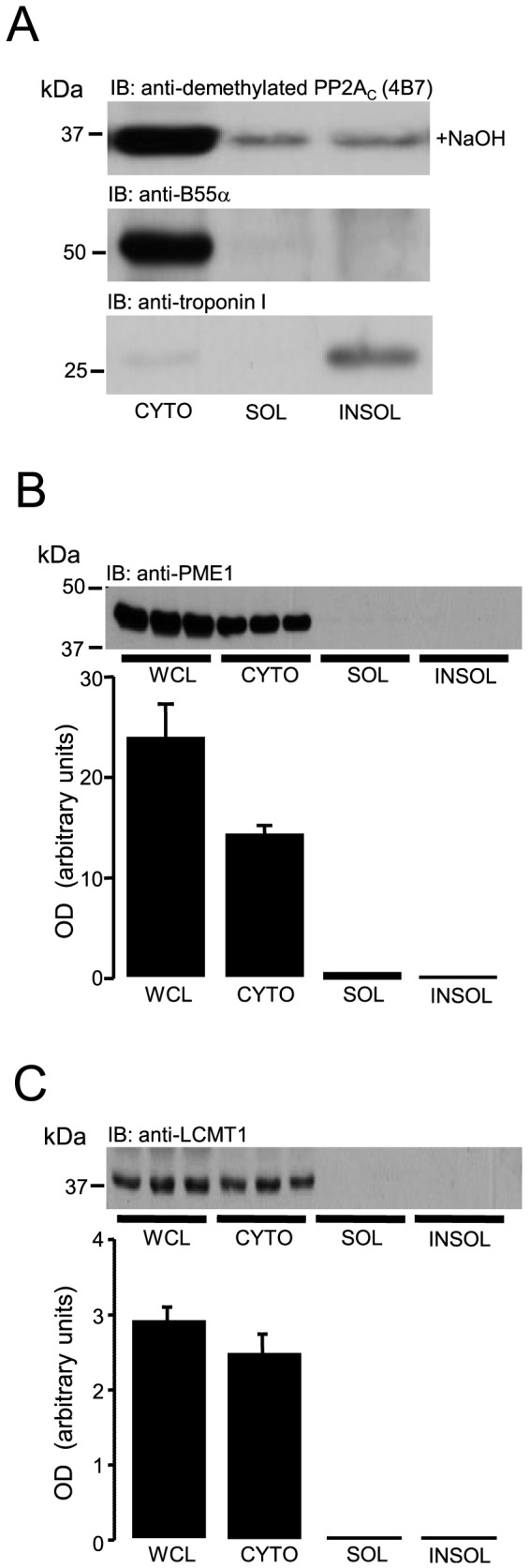
Subcellular localisation of proteins that regulate PP2A_C_ carboxylmethylation and targeting in ARVM. ARVM were initially lysed with a digitonin-based buffer to separate the cytoplasm and particulate fractions. The particulate fraction was then resuspended with a Triton X-100 based buffer to separate the soluble membrane and insoluble fractions by centrifugation. Samples were then analysed by standard Western immunoblotting (IB). (A) Subcellular localisation of PP2A_C_ and B55α protein in the cytoplasm (CYTO), soluble membrane (SOL) and insoluble (INSOL) fractions in ARVM. The presence of cardiac troponin I (cTnI) protein was to confirm the purity of the insoluble (INSOL) fraction. Subcellular localisation of PME-1 (B) and LCMT-1 (C) protein in the whole cell lysate (WCL), cytoplasm (CYTO), soluble membrane (SOL) and insoluble (INSOL) fractions in ARVM. All columns represent mean optical density (OD) values ± SEM, n = 3 individual experiments.

We initially aimed to determine the subcellular localisation of components comprising the LCMT-1/PME-1/PP2A signalling axis in ARVM. Previous studies have demonstrated that LCMT-1 protein expression in non-myocytes is restricted to the cytoplasm, whereas PME-1 has been shown to be predominantly expressed in the nucleus [35]. The subcellular fractionation of ARVM by digitonin/Triton X-100 based lysis is shown in Fig. 1 and confirms our own previous observation [23] that the PP2A_C_ subunit is predominantly localised to the cytoplasmic fraction of ARVM (Fig. 1A). Furthermore, Fig. 1A also shows that B55α (PPP2R2A) regulatory subunit isoform that associates with the PP2A_A/C_ heterodimer in a methylation dependent manner [8] is similarly localised to the cytoplasmic fraction of ARVM. Importantly, both PME-1 (Fig. 1B) and LCMT-1 (Fig. 1C) protein expression appears to be almost exclusively localised in the cytoplasm of ARVM, with negligible localisation in the soluble membrane and insoluble fractions. This suggests that all components thought to regulate the carboxylmethylation and methylation-dependent targeting of PP2A_C_ to cellular substrates are predominantly localised in the cytoplasm of ARVM.

### G_i_PCR-induced PP2A_C_ carboxylmethylation in adult rat ventricular myocytes

**Figure 2:**
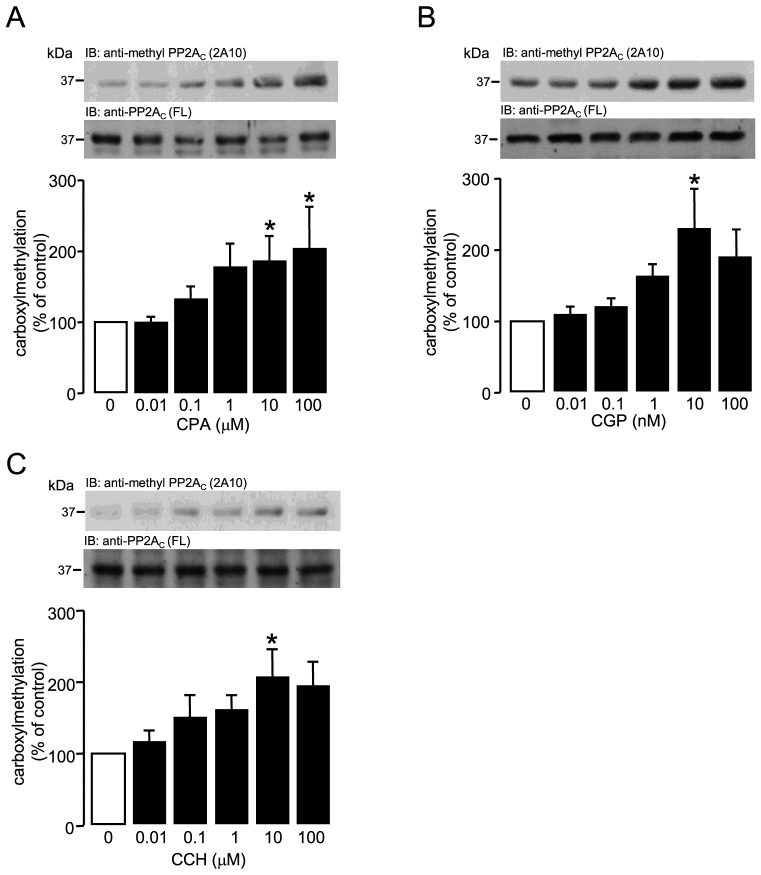
G_i_PCR-induced carboxylmethylation of PP2A_C_ in ARVM. ARVM were exposed to the G_i_PCR agonists (A) CPA (0–100 µM), (B) CGP (0–100 nM) or (C) CCH (0–100 µM) for 10 minutes. Carboxylmethylation of PP2A_C_ (as % of vehicle control) was detected by Western immunoblotting (IB) of the whole cell lysate with either an anti-methyl PP2A_C_ (2A10) or an anti-PP2A_C_ FL-309 (FL) antibody to determine equal protein loading. PP2A_C_ carboxylmethylation was quantified by densitometry and all columns represent mean values ± SEM, n = 4–6 individual experiments, *p<0.05 vs 0 (control group).

Using an anti-methyl PP2A_C_ antibody (2A10) to detect the methylation status of the C-terminal leu309 of PP2A_C_, data shown in Fig. 2A extends previously reported observations [22]–[24] that the stimulation of G_i_-protein coupled A1.Rs by N6-cylcopentyladenosine (CPA) induces carboxylmethylation of PP2A_C_, by establishing the concentration-dependence of the response. In addition, we provide evidence shown that the Angiotensin II type 2 receptor (AT_2_) agonist CGP42112 (Fig. 2B) and the muscarinic receptor agonist carbachol (Fig. 2C) also induce PP2A_C_ carboxylmethylation in a concentration-dependent manner. Equal protein loading was determined by using a polyclonal anti-PP2A_C_ (FL-309) to detect both non-methylated and methylated forms of PP2A_C_. These data suggest that PP2A_C_ carboxylmethylation can be induced not only by the stimulation of A1.Rs but also by the stimulation of other G_i_PCRs.

**Figure 3:**
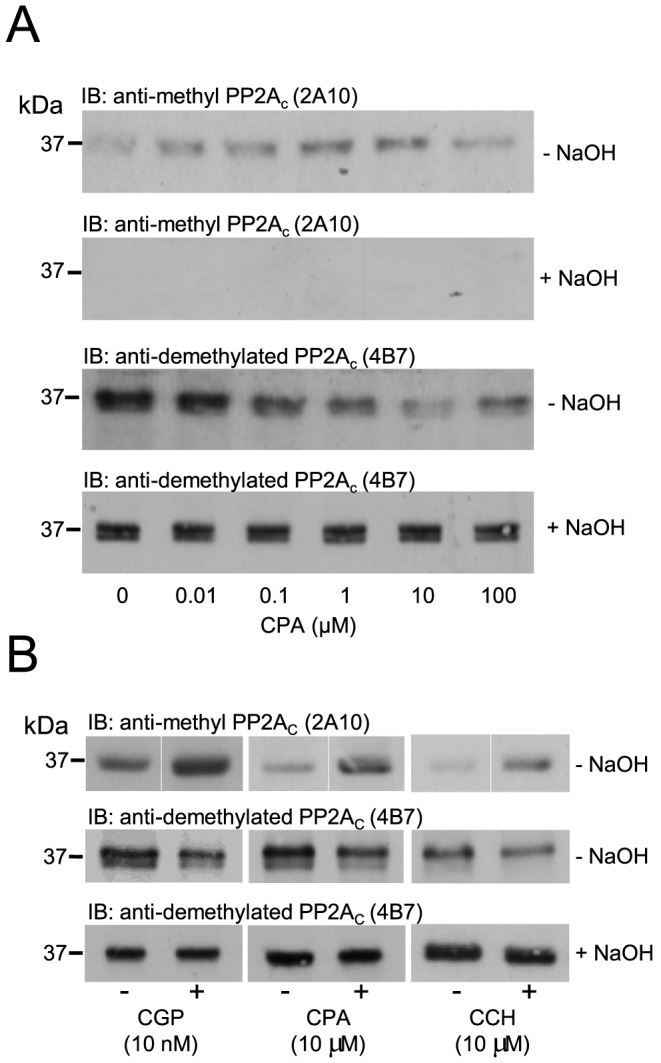
Saponification of carboxylmethylated PP2A_C_ by alkalinisation. ARVM were exposed to the A1.R agonist CPA (0–100 µM) for 10 minutes and PP2A_C_ carboxylmethylation was detected by Western immunoblotting (IB) with either an anti-methyl PP2A_C_ (2A10) or demethylated PP2A_C_ (4B7) antibody before (−NaOH) or after (+NaOH) saponification of the C-terminal leu309 methylation by 100 mM NaOH. (B) Carboxylmethylation of PP2A_C_ in response to CGP (10 nM), CPA (10 µM) or CCH (10 µM) as detected by Western immunoblotting with either an anti-methyl PP2A_C_ (2A10) or demethylated PP2A_C_ (4B7) antibody with or without treatment with 100 mM NaOH. Immunoblots are representative of 3 individual experiments.

Using NaOH to remove the methyl moiety from the C-terminal leu309 of PP2A_C_ by saponification, we were able to demonstrate that the anti-methyl PP2A_C_ antibody (2A10) was no longer able to detect carboxylmethylated PP2A_C_. Methylation of leu309 within the TPDYFL C-terminal tail of PP2A_C_ masks the 4B7 antibody epitope and sterically interferes with antibody recognition of PP2A_C_ protein. We show in Fig. 3A that A1.R stimulation by CPA induces an increase in PP2A_C_ carboxylmethylation, which is confirmed by two different antibodies raised against carboxylmethylated PP2A_C_ (2A10) or demethylated PP2A_C_ (4B7). Having used NaOH to remove the C-terminal (leu309) methyl moiety, the antibody raised against demethylated PP2A_C_ (4B7) showed an equal amount of total PP2A_C_ in all protein sample lanes. We believe that this constitutes an effective protocol for the determination of total PP2A_C_ in any protein sample. Fig. 3B confirms that the increased level of PP2A_C_ carboxylmethylation in response to either CGP42112 or carbachol is also sensitive to saponification by NaOH.

### Effects of CPA on cellular PP2A_C_ binding partners

**Figure 4:**
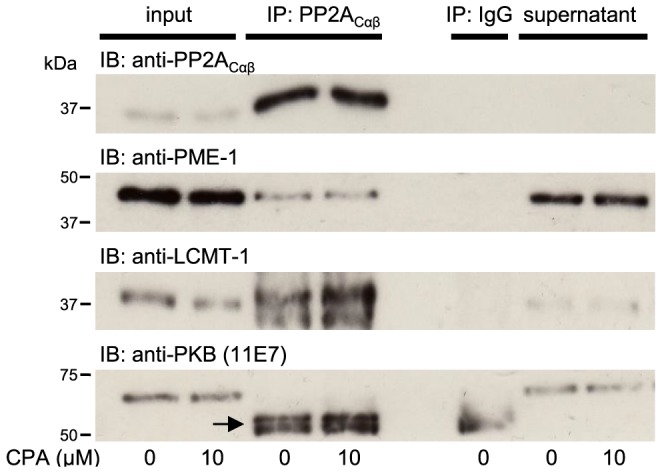
Effects of CPA on the association of PP2A_C_ with cellular binding partners. ARVM were exposed to either vehicle (0.1% DMSO) control (0) or the A1.R agonist CPA (10 µM) for 10 minutes followed by lysis with an immunoprecipitation buffer. PP2A_Cα/β_ was then immunoprecipitated from the resulting ARVM lysates and the immunocomplexes were then probed for the presence of PP2A_Cα/β_, PME-1, LCMT-1 and PKB by standard Western immunoblotting. Immunoblots show the levels of the relevant proteins in the input, PP2A_Cα/β_ immunocomplexes (IP: PP2A_Cα/β_), non-specific immunocomplexes (IP: IgG) and supernatant (post-immunoprecipitation). The black arrow denotes detection of the immunoprecipitating IgG molecules.

Several studies have shown that PP2A_C_ can associate with several regulatory proteins such as PME-1 [14], LCMT-1 [16] and PKB [36] in non-myocytes. In support of this data, figure 4 shows that PP2A_C_ does indeed exist in association with both PME-1 and LCMT-1 in unstimulated adult rat ventricular myocytes. Interestingly the data also demonstrate that although the exposure of ARVM to CPA does not alter the association between PP2A_C_ and PME-1, it increases the association between PP2A_C_ and LCMT-1. The data also suggest that, unlike in other cell types [36], there is no apparent association between cellular PP2A_C_ and PKB in adult rat ventricular myocytes.

### Role of Gβγ subunits in CPA-induced PP2A_C_ translocation in adult rat ventricular myocytes

**Figure 5:**
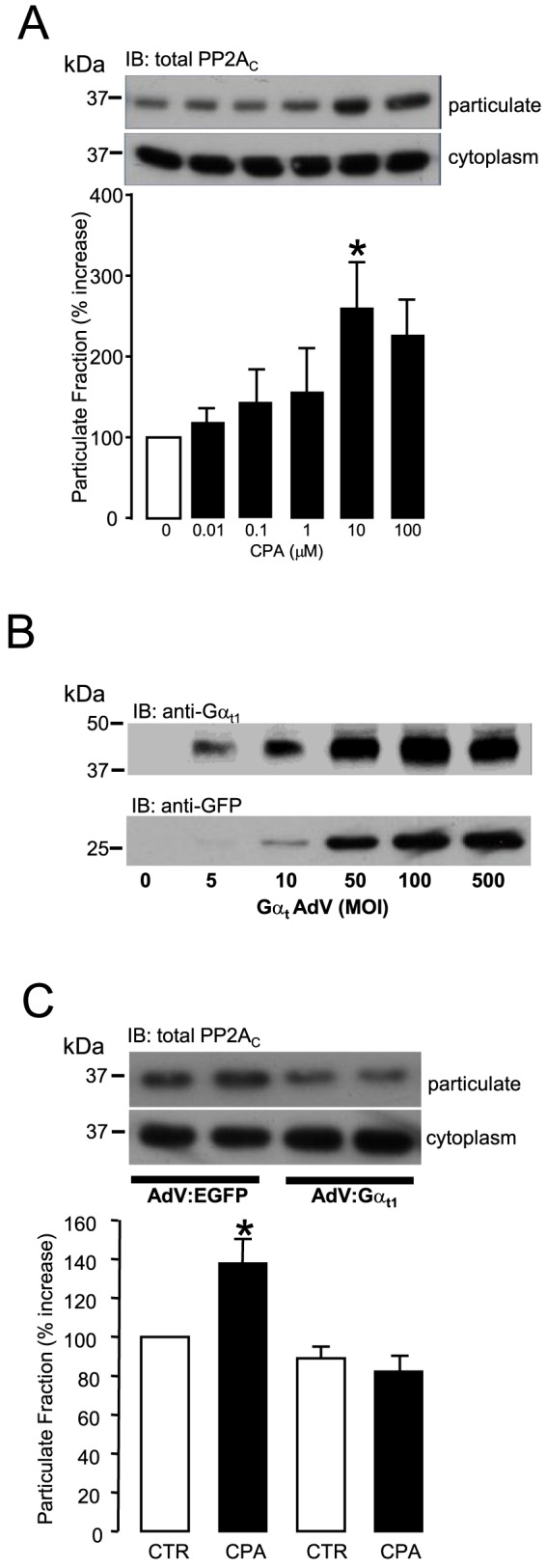
Role of Gβγ subunits in CPA-induced PP2A_C_ translocation. ARVM were lysed with a digitonin-based buffer to separate the cytoplasm and particulate fractions by centrifugation. Samples were then saponified with NaOH to abrogate any PP2A_C_ carboxylmethylation. (A) Total PP2A_C_ content in the particulate fraction of ARVM in response to increasing concentrations of CPA (0–100 µM) was indexed by Western immunoblotting (IB) with an anti-demethylated PP2A_C_ antibody (4B7) following treatment with 100 mM NaOH. Total PP2A_C_ content in the particulate fraction was quantified by densitometry. (B) Multiplicity of infection (MOI)-dependent co-expression of EGFP and Gα_t1_ protein in ARVM infected with the AdV:Gα_t1_. (C) ARVM were infected with either the control AdV:EGFP or AdV:Gα_t1_ for 18 hours and then exposed to10 µM CPA for 10 minutes. ARVM were then lysed and total PP2A_C_ content in the particulate fraction was indexed by Western immunoblotting (IB) with an anti-demethylated PP2A_C_ (4B7) antibody following treatment of samples with 100 mM NaOH. PP2A_C_ content in the particulate fraction was quantified by densitometry. All columns represent mean values ± SEM, n = 4 individual experiments, *p<0.05 vs 0 (control group).

The classical effect of G_i_PCR stimulation is to induce Gα_i_-mediated inhibition of membrane bound adenylate cyclase [37], however, Gβγ subunits are known to mediate a range of intracellular signalling events of their own [38]. In this study we have exposed ARVM to increasing concentrations of CPA (0–100 µM) for 10 minutes and determined the translocation of PP2A_C_ to the membrane-rich particulate fraction. Figure 5A shows that CPA significantly (p<0.05) increased the total PP2A_C_ content within the particulate fraction of ARVM in a concentration dependent manner. To test the hypothesis that Gβγ subunits were mediators of the intracellular signalling cascade that led to G_i_PCR-induced PP2A_C_ translocation to the particulate fraction of ARVM in response to G_i_PCR stimulation, we adenovirally expressed the Gα subunit of the ocular transducin GPCR, Gα_t1_ in these cells. Several studies have demonstrated that heterologous expression of the Gα_t1_ subunit in cells can sequester Gβγ subunits and switch off Gβγ subunit-dependent intracellular signalling [27], [39], [40]. Figure 5B illustrates the MOI-dependent levels of EGFP and Gα_t1_ protein co-expression and indicates that considerable heterologous expression of Gα_t1_ protein was achieved at an MOI of 50 pfu/ARVM. Hence, ARVM were infected with either a control EGFP-expressing or Gα_t1_-expressing adenovirus (each at 50 MOI) and then exposed to 10 µM CPA for 10 minutes. Figure 5C shows that CPA-induced significant (p<0.05) PP2A_C_ translocation to the particulate fraction in the presence of EGFP expression. However, this translocation was abrogated in ARVM infected to express Gα_t1_ in order to sequester Gβγ subunits. These data suggest that CPA-induced PP2A_C_ translocation to the particulate fraction of ARVM is mediated by Gβγ subunits.

### CPA-induced activation of phosphoinositide 3-kinase (PI3K) and redistribution of PP2A_C_ in adult rat ventricular myocytes

**Figure 6:**
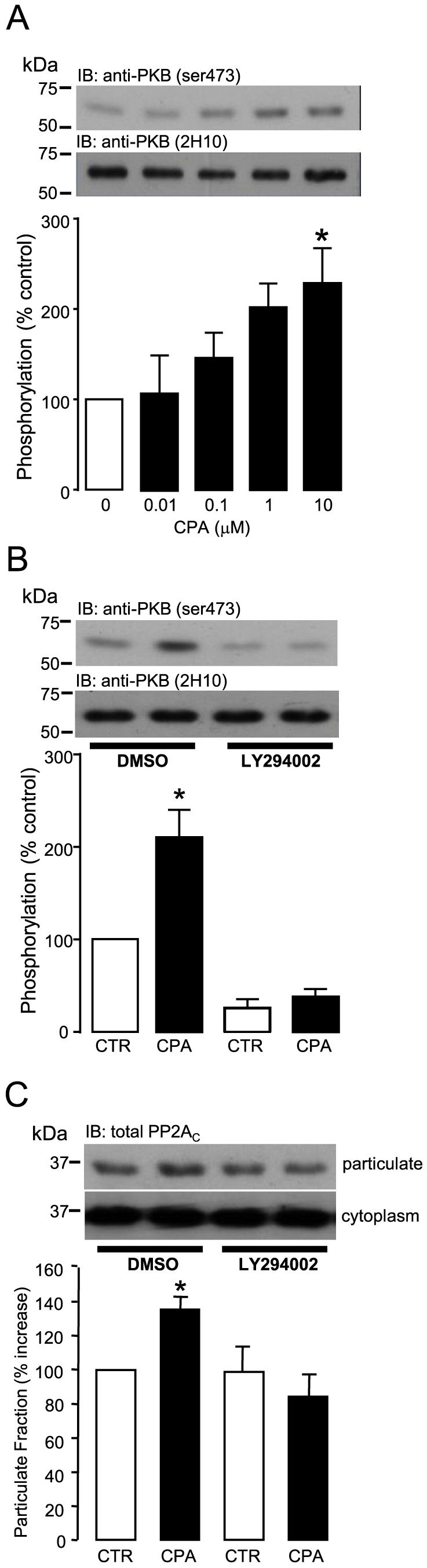
Role of PI3K in CPA-induced PP2A_C_ translocation. (A) ARVM were exposed to the A1.R agonist CPA (0–100 µM) for 10 minutes and then lysed with SDS-PAGE Laemmli sample buffer for the determination of PKB phosphorylation (ser473) and total PKB by Western immunoblotting (IB). PKB (ser473) phosphorylation was quantified by densitometry. (B) ARVM were pretreated with either 0.1% DMSO or 20 µM LY294002 for 30 minutes and then exposed to 0.1% DMSO (CTR) or 10 µM CPA for 10 minutes. PKB phosphorylation (ser473) and total PKB were then determined by Western immunoblotting (IB). PKB (ser473) phosphorylation was quantified by densitometry. (C) ARVM were pretreated with either 0.1% DMSO or 20 µM LY294002 for 30 minutes and then exposed to 0.1% DMSO (CTR) or 10 µM CPA for 10 minutes. ARVM were then lysed with 0.05% digitonin and fractionated by centrifugation. Samples were then saponified with NaOH to remove any PP2A_C_ carboxylmethylation. Total PP2A_C_ content in the particulate fraction of ARVM was indexed by Western immunoblotting (IB) with anti-demethylated PP2A_C_ (4B7) antibody following treatment with 100 mM NaOH. Total PP2A_C_ content in the particulate fraction was quantified by densitometry. All columns represent mean values ± SEM, n = 4 individual experiments, *p<0.05 vs 0 (control group).

Several studies have shown that G_i_PCR stimulation can activate the PI3K family of lipid/protein kinases [41]–[43]. To determine that this occurs under our experimental conditions, we used PKB (ser473) phosphorylation status as a surrogate index of PI3K activity. CPA was found to induce significant (p<0.05) phosphorylation of PKB at ser473 (Fig. 6A) and pretreatment of ARVM with the PI3K inhibitor LY294002 (20 µM) abrogated this response (Fig. 6B). These observations suggest that not only did the activation of G_i_-coupled A1.Rs induce the phosphorylation of PKB (ser473), but that this occurred in a PI3K-dependent manner in ARVM. Importantly, Fig. 6C shows that pretreatment of ARVM with LY294002 (20 µM) abrogated CPA-induced PP2A_C_ translocation to the particulate fraction of ARVM, thereby suggesting that G_i_PCR-induced PP2A_C_ translocation is dependent on PI3K activation in ARVM.

## Discussion

Several studies [22]–[24] have previously shown that the subcellular distribution of PP2A_C_ is predominantly restricted to the cytoplasm of ARVM, which we confirmed in the current study. Furthermore, the PP2A_C_ regulatory B subunit (PPP2R2A/B55α) which is known to associate exclusively with carboxylmethylated PP2A_C_ [8] and form targetable PP2A heterotrimers, was also restricted to the cytoplasm of ARVM (Fig. 1A). This confirms that both PP2A_C_ and B55α are localised to the same cellular compartment of ARVM. The overall cellular carboxylmethylation status of PP2A_C_ is thought to reflect the balance in activity of LCMT-1 and PME-1 towards their common substrate PP2A_C_. X-ray crystallographic data suggest that the active sites of PP2A_C_, LCMT-1 [16] and PME-1 [14] can interact with each other to form tight PP2A_C_/LCMT-1 and PP2A_C_/PME-1 complexes, which regulate the methylation status of the C-terminal leu309 of PP2A_C_. These observations are supported by our own novel finding in ARVM, which suggest that cellular PP2A_C_ does indeed basally form an association with both PME-1 and LCMT-1. Assuming that the activities of PME-1 and LCMT-1 remain constant, the increase in C-terminal leu309 PP2A_C_ methylation as a result of G_i_PCR stimulation, may be explained either by a reduced PME-1 or an increased LCMT-1 association with PP2A_C_. Our data suggests that the latter may be true as G_i_PCR stimulation does not affect the association of PP2A_C_ with PME-1 protein. This novel observation may explain the basis by which G_i_PCR stimulation induces an increase in C-terminal leu309 carboxylmethylation of PP2A_C_. Interestingly, immunoprecipitation of PP2A_C_ removed virtually all PP2A_C_ protein from the lysate (to undetectable levels) and a significant proportion of cellular LCMT-1 protein was immunoprecipitated along with it. Hence, the pool of cytoplasmic PP2A_C_ in this complex may represent a reservoir of predominantly carboxylmethylated PP2A_C_ subunits. Although PME-1 protein was shown to associate with PP2A_C_, a significant proportion of PME-1 protein was still present in the immunocomplex supernatant (post-immunoprecipitation) and it is likely that the PP2A_C_ in this complex is predominantly in the demethylated state [9], [10].

As PP2A_C_ subunits are the only known LCMT-1/PME-1 substrates, it is possible that cytoplasmic PP2A_C_ restricts the localisation of LCMT-1, and to a lesser extent PME-1 protein, in the cytoplasm. PME-1 protein in human HeLa cells contains a functional 270KRKK273 nuclear localisation sequence (NLS), which explains why in this cell type PME-1 protein is thought to be predominantly localised in the nucleus together with demethylated PP2A_C_ [35]. The human and rat PME-1 proteins are highly (98%) homologous, however, much of the variance that exists between these two PME-1 proteins, does so in the residues adjacent to the NLS. Rat PME-1 protein contains the sequence 268VNKRKK273 which differs to the human PME-1 protein sequence 268ISKRKK273. It is possible that the NLS contained within rat PME-1 protein is in some way dysfunctional due to the preceding valine/asparagine residues, which may partly explain why in ARVM, PME-1 protein is predominantly found in the cytoplasm and not the nucleus.

The carboxylmethylation and subsequent translocation of PP2A_C_ to the particulate fraction, in response to stimulated G_i_ protein coupled A1.Rs, within ARVMs was reported several years ago by the Hofmann laboratory [22], [24], [44]. In these studies, the phosphorylation of proteins in response to isoprenaline, present in the particulate fraction (troponin I and phospholamban), was found to be decreased by the stimulation of A1.Rs in a phosphatase-dependent manner. Our previous studies not only confirmed these observations, but identified an additional membrane bound protein (Na^+^/H^+^ exchanger isoform-1) as a novel PP2A_C_ substrate and demonstrated that the translocation of PP2A_C_ to the particulate fraction was sensitive to pertussis toxin [23]. Despite these studies suggesting that PP2A_C_ can be regulated by G_i_PCR stimulation, very little has been reported since regarding the cellular mechanisms involved in the regulation of PP2A_C_ by inhibitory class GPCRs. A possibility that the PP2A_C_ translocation observed in ARVM may be A1.R-specific did exist. Hence, in this study we also chose to use other G_i_PCR agonists such as carbachol and CGP42112 to target M_2_.Rs and AT_2_.Rs, respectively. Our data show that the stimulation of M_2_.Rs and AT_2_.Rs also induced PP2A_C_ carboxylmethylation in a concentration-dependent manner. The data suggests that PP2A_C_ carboxylmethylation is not a unique consequence of A1.R stimulation but can occur downstream of other G_i_PCRs. To confirm that PP2A_C_ carboxylmethylation in ARVM following exposure to CPA, carbachol or CGP42112 as detected by the anti-methyl PP2A_C_ antibody was genuine, we used NaOH to remove the methyl moiety by saponification from the C-terminal leu309 of PP2A_C_ [23], [45], [46]. Saponification abolished the signal detected by the anti-methyl PP2A_C_ antibody and equalised the signal in all lanes, as detected by the anti-demethylated (4B7) PP2A_C_ antibody. This confirmed that the methyl moiety on the C-terminal leu309 of PP2A_C_ conferred antibody epitope masking as previously reported [23], [45], [46].

These novel observations led us to next investigate mechanisms downstream of G_i_PCR stimulation and we focused our attention on the role of Gβγ subunits in G_i_PCR-induced cellular PP2A_C_ redistribution. We chose to sequester Gβγ subunits by adenovirally expressing the Gα_t1_ subunit of the ocular transducin GPCR, a protein not natively expressed in ARVM. Several studies [27], [39], [47] have successfully expressed Gα_t1_ subunits to implicate a role for Gβγ subunits in intracellular signalling events. Our data demonstrate that heterologous expression of Gα_t1_ subunits abrogated G_i_PCR-induced PP2A_C_ translocation to the particulate fraction of ARVM. This is supported by studies suggesting the existence of a G_i_PCR (A1.R)-Gβγ signalling hub in cardiac tissue. The stimulation of A1.Rs by chlorocyclopentyl adenosine in murine hearts was shown to activate phospholipase C in a Gβγ-dependent manner [48]. Furthermore, the stimulation of the dual (G_s_/G_i_) coupled β_2_-adrenoceptor (β_2_.AR) was reported to promote the survival of adult mouse cardiomyocytes following exposure to isoprenaline. In this study, the carboxyl terminus of β-adrenoreceptor kinase (βARK-ct) a commonly used Gβγ inhibitor [49] abrogated isoprenaline-induced PKB activation and cellular survival, thereby implying that β_2_.AR/G_i_-induced cardiomyocyte survival involves Gβγ subunit activation [41]. Our novel data suggests that a proximal signalling step linking G_i_PCRs to the regulation of PP2A_C_ cellular localization is mediated by Gβγ subunits.

Evidence [41], [50] suggests that Gβγ subunits initiate intracellular signalling cascades via the activation of PI3K [38]. It has been considered for some time that class IB PI3Ks can be activated by Gβγ dimers [51], [52]. Class IB PI3Ks consist of a p110γ catalytic subunit associated with a regulatory subunit referred to as p101 [51]. The regulatory subunit p101 is central to the Gβγ-mediated activation of class IB PI3Ks [52]. Our observations with the PI3K inhibitor LY294002 suggest that A1.R stimulation induces PP2A_C_ translocation to the particulate fraction via a Gβγ/PI3K signalling axis. In support of our observations, Zhu *et al* [41] reported that the G_i_ arm of the dual G_s_/G_i_-coupled β_2_-adrenoceptor could also activate PI3K via Gβγ subunits in murine cardiomyocytes. Hence our data suggests that A1.Rs are linked to PP2A_C_ by a Gβγ/PI3K signalling axis.

**Figure 7:**
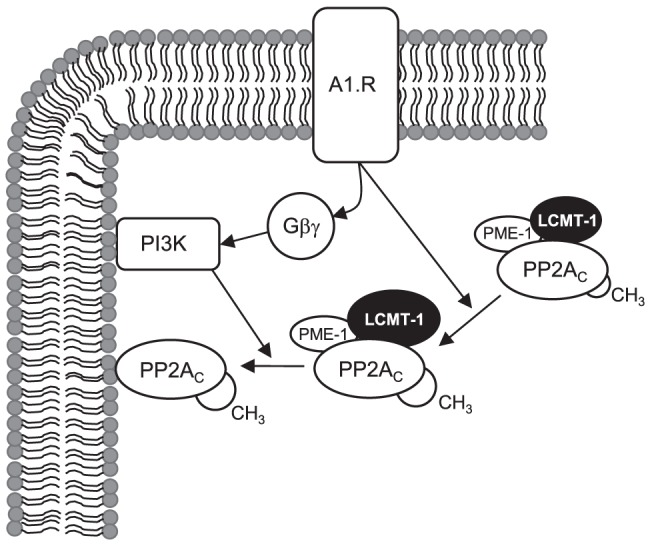
Suggested intracellular signalling mechanism (s) through which A1.Rs induce PP2A_C_ translocation. Our data suggests that the stimulation of G_i_ protein-coupled adenosine A1 receptors by the agonist CPA increases the association of PP2A_C_ with LCMT-1, thereby augmenting the leucine carboxylmethylation status of PP2A_C_. The stimulation of G_i_ protein-coupled adenosine A1 receptors by the agonist CPA also elicits a cascade involving the release of Gβγ subunits which activate PI3K. Both of these intracellular signalling events coordinate and facilitate the association of PP2A_C_ with the membrane-rich particulate compartment of ARVM.

In conclusion, our data indicate that G_i_PCR agonists may induce the carboxylmethylation of PP2A_C_ by increasing its association with the methyltransferase LCMT-1. G_i_PCR agonists also mediate the disassociation of Gα_i_βγ heterotrimeric proteins and the released Gβγ subunits in turn activate PI3K. The simultaneous activation of PI3K and induction of PP2A_C_ carboxylmethylation appear to coordinate the translocation of PP2A_C_ to the particulate fraction of ARVM in response to G_i_PCR stimulation (see figure 7).
